# A gene expression signature shared by human mature oocytes and embryonic stem cells

**DOI:** 10.1186/1471-2164-10-10

**Published:** 2009-01-08

**Authors:** Said Assou, Doris Cerecedo, Sylvie Tondeur, Véronique Pantesco, Outi Hovatta, Bernard Klein, Samir Hamamah, John De Vos

**Affiliations:** 1 CHU Montpellier, Institute for Research in Biotherapy, Hôpital Saint-Eloi, Montpellier, F-34000 France; 2 INSERM, U847, Montpellier, F-34000 France; 3 Université MONTPELLIER1, UFR de médecine, Montpellier, F-34000 France; 4 MacoPharma, Tourcoing, F-59200 France; 5 Department of Obstetrics and Gynecology, CLINTEC, Karolinska Institutet, Karolinska University Hospital, Huddinge, Stockholm, Sweden; 6 CHU Montpellier, Unité biologie clinique d'AMP – DPI, Hôpital Arnaud de Villeneuve, Montpellier, F-34000 France

## Abstract

**Background:**

The first week of human pre-embryo development is characterized by the induction of totipotency and then pluripotency. The understanding of this delicate process will have far reaching implication for in vitro fertilization and regenerative medicine. Human mature MII oocytes and embryonic stem (ES) cells are both able to achieve the feat of cell reprogramming towards pluripotency, either by somatic cell nuclear transfer or by cell fusion, respectively. Comparison of the transcriptome of these two cell types may highlight genes that are involved in pluripotency initiation.

**Results:**

Based on a microarray compendium of 205 samples, we compared the gene expression profile of mature MII oocytes and human ES cells (hESC) to that of somatic tissues. We identified a common oocyte/hESC gene expression profile, which included a strong cell cycle signature, genes associated with pluripotency such as *LIN28 *and *TDGF1*, a large chromatin remodelling network (*TOP2A, DNMT3B, JARID2, SMARCA5, CBX1, CBX5*), 18 different zinc finger transcription factors, including *ZNF84*, and several still poorly annotated genes such as *KLHL7*, *MRS2*, or the Selenophosphate synthetase 1 (*SEPHS1*). Interestingly, a large set of genes was also found to code for proteins involved in the ubiquitination and proteasome pathway. Upon hESC differentiation into embryoid bodies, the transcription of this pathway declined. In vitro, we observed a selective sensitivity of hESC to the inhibition of the activity of the proteasome.

**Conclusion:**

These results shed light on the gene networks that are concurrently overexpressed by the two human cell types with somatic cell reprogramming properties.

## Background

Oocytes have the unique ability to remodel the chromatin of the germinal nuclei into a totipotent state. These modifications are particularly striking for the male pro-nuclei: upon fertilization, the sperm chromatin packaging protamines are stripped off and replaced by histones, the DNA is demethylated within 4 hours of fertilization, and the amino terminal tails of histones are modified including methylation of arginin 9 and phosphorylation of serin 10 of histone H3 (*H3K9 *and *PhH3S10*, respectively) [1,2]. Remarkably, the reprogramming properties of oocytes are not restricted to the very specialized germinal nuclei. Indisputably, the cloning of Dolly has shown that the oocyte cytoplasm is able to extensively reverse the chromatin modifications associated with a differentiated state [3,4]. Somatic cell nuclear transplantation (SCNT) has since been extended to other species, including human cells, and to many cell types, including terminally differentiated cells such as granulocytes [5,6]. Thus differentiation is not anymore considered as an irreversible process, but rather as modifications of the cellular epigenome and transcriptome, that are amenable to complete reversal. In addition to oocytes, other cell types can reprogram somatic cells towards pluripotency. For example, using cell fusion strategies, it has been shown that hybrid cell clones obtained by fusion of a differentiated cell with either teratocarcinoma cells or embryonic stem cells display features of pluripotent, undifferentiated cells with concomitant loss of the markers associated with differentiation [7,8]. More recently, and quite unexpectedly, Takahashi and Yamanaka have shown that the expression of only four selected transcription factors, *OCT3/4*, *SOX2*, *CMYC *and *KLF4*, is sufficient to drive a mouse fibroblast into an induced pluripotent stem cell (iPS) with all the features of embryonic stem cells, including a high growth rate and the ability to form a variety of tissues from all three germ layers in vitro and in vivo [9]. These results have been confirmed by other studies, extended to human cells, and applied to non-fibroblastic cells such as mesenchymal stem cells (MSCs), gastric epithelial cells or hepatocytes [10-12]. At the center of cellular reprogramming lies the activation of the pluripotency transcriptional regulatory circuitry involving *POU5F1/OCT4*, *NANOG *and SOX2 [13] and extensive chromatin-remodeling. However, the details of this process, such as the exact mediators of the chromatin modifications, remain ill defined. Data from xenopus egg experiments point to nucleosomal ATPases, but these findings await confirmation using mammalian oocytes [14,15].

As oocytes and ES cells are two cell types able to reprogram a somatic cell such as fibroblasts into pluripotent cells, the comparison of the gene expression program of these two cell types could contribute to the understanding of these cell reprogramming properties. Therefore, we generated a transcriptome compendium of 205 samples by collecting public microarray data and compared the gene expression profile of oocytes and hESC to that of somatic tissues. We defined a common oocyte/hESC signature, which comprised many cell cycle genes, but also several biological pathways not associated with cell growth. Strikingly, a large set of genes is coding for genes involved in protein ubiquitination and the proteasome pathway. Upon hESC differentiation into embryoid bodies, the transcription of this pathway declines. In agreement with this preferential expression in pluripotent cells, we observed a selective sensitivity of hESC to the pharmacological inhibition of the proteasome activity, suggesting a role for this machinery in the maintenance of pluripotency.

## Results

### Human oocytes and hESC share a common transcriptome signature

**Figure 1:**
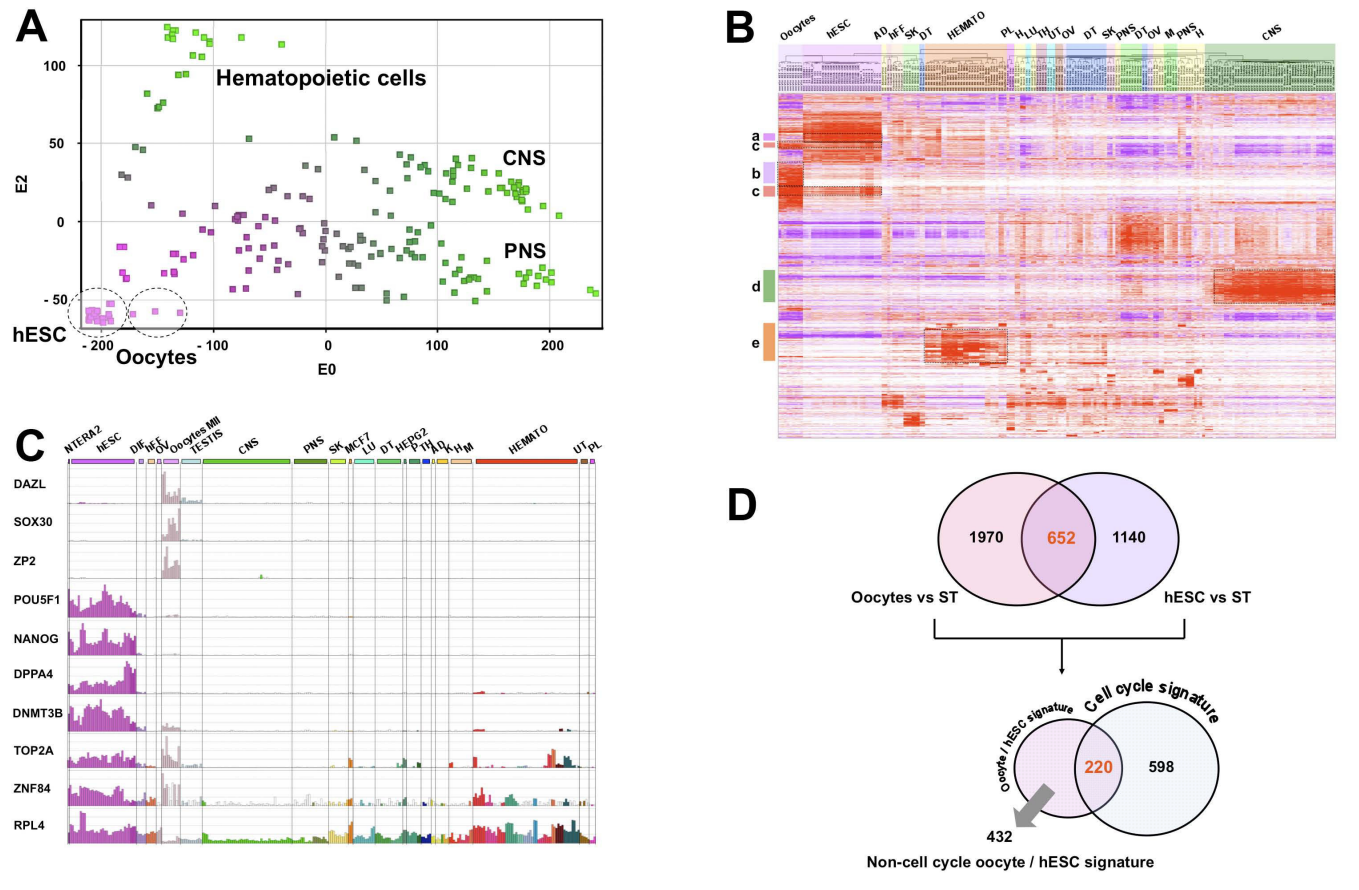
**Human mature MII oocytes and hESC display a common transcriptome signature**. Principal component analysis (PCA) (A). A PCA of 9 mature oocytes (MII), 29 hESCs and 167 somatic tissue samples. CNS, central nervous system; PNS, peripheral nervous system. Hierarchical clustering (B). Each horizontal line represents a PS and each column represents a single sample. PS up and down-regulated were colored in red and purple, respectively. In white, PS which were neither over nor under-expressed. Clusters: a: hESCs genes; b: MII oocytes genes; c: the oocytes/hESC signature; d: genes expressed in CNS; e: genes expressed in hematopoietic cells. AD: adipocytes; hFF: human foreskin fibroblasts; SK: skin and keratinocytes; DT: digestive tract; Hemato: hematopoietic cells; PL: placenta; H: heart; LU: lung; TH: thyroid; UT: uterus; OV: ovary; M: muscle. Expression bar charts of selected genes (C). Expression bar charts of three oocytes specific genes (DAZL, SOX30, ZP2), three hESCs specific genes (*POU5F1/OCT4, NANOG, and DPPA4*), three genes up-regulated in hESC and oocytes MII (*DNMT3B, TOP2A and ZNF84*) and one ubiquitously expressed gene (*RPL4*) using our on-line expression atlas Amazonia! . Diff: non-specific differentiated hESC; MCF7: breast cancer cell line; HEPG2: hepatocarcinoma cell line. Venn diagram detailing shared and distinct gene expression among hESCs and MII human oocytes (D). The oocyte/hESC signature was defined as the intersection of the hESC signature (genes overexpressed in hESC compared to somatic tissues (ST)) and the MII oocytes signature (genes overexpressed in MII oocytes compared to ST). The oocytes/hESC signature was then further parted into a fraction sharing a cell cycle signature and a non-cell cycle part.

To identify a gene expression signature shared by mature oocytes and hESC but not by somatic cells, we confronted 9 oocyte and 29 hESC expression profiles to a transcriptome collection of 167 samples spanning a wide variety of fetal and adult somatic cell samples (see Table 1; see Additional file 1). This microarray compendium quantified the expression of 13 279 unique Refseq transcripts in a total of 205 samples (see Methods and Additional file 2). A non-supervised analysis using principal component analysis (PCA) mapped the samples in a two dimensional space with a differentiation gradient ranging from undifferentiated hESC samples to highly specialized tissues such as hematopoietic cell samples or nervous system tissue samples (Figure 1A). The hESC samples grouped together very tightly, even though the transcriptomes were obtained from six different studies and included 15 different hESC cell lines, in agreement with the low variation between hESC cell lines [16] and the robustness of the Affymetrix microarrays [17]. Strikingly, the human mature oocyte samples were situated in the vicinity of the hESC samples, distantly located from most somatic tissues samples. A hierarchical clustering confirmed these findings, showing that MII oocytes and hESC clustered together, sharing a signature of overexpressed genes, demonstrating a close gene expression (Figure 1B; see Additional file 3). We computed an oocyte and a hESC signature by comparing each of these two categories to somatic cells. Using Significance Analysis of Microarrays (SAM) software with a false discovery rated (FDR) of 0.05% and a ratio between groups of at least 2, we determined that oocytes overexpressed 2622 probesets (PS) (2097 different Refseq transcripts) compared to somatic samples, whereas hESC overexpressed 1792 PS (1436 different Refseq transcripts) (see Additional file 4). The "oocytes signature" comprised *DAZL*, *SOX30*, *ZP2*, *GDF9*, *AURKC*, *PTTG3*, etc. (Figure 1C) which have previously been identified as overexpressed by female germinal cells by our group and others [18,19]. Similarly, the "hESC signature" displayed numerous genes known to be specifically overexpressed in hESC such as *POU5F1/OCT4*, *NANOG*, *DPPA4*, *TDGF1*, *CD24*, *PODXL*, *HELLS*, etc. [20]. These findings validated the biological relevance of our compendium and the signatures specific to oocytes and hESC. We intersected these two lists of genes and established a "oocyte/hESC signature", composed of 652 PS (558 different Refseq transcripts) (Figures 1D; see Additional file 4).

**Table 1:** Samples of the microarray compendium

**Cell type**	**Tissue or cell line**	**Normal or Malignant**	**Number of samples**	**References**
**Normal cell compendium (n = 205)**				

Human embryonic stem cells	Cell line	Normal	29	[38-43]

Mature oocytes	Cells	Normal	9	[18,44,45] and this study

Foreskin fibroblasts	Cell line	Normal	4	[40]

Ovary	Tissue	Normal	3	[46,47]

Central nervous system	Tissue	Normal	44	[46,47]

Peripheral nervous system	Tissue	Normal	18	[46,47]

Skin and keratinocytes	Tissue and cultured primary cells	Normal	6	[39]

Lung	Tissue	Normal	11	[46,47]

Digestive tract	Tissue	Normal	19	[46,47]

Thyroid	Tissue	Normal	5	[46,47]

Adipocytes	Tissue	Normal	2	[46,47]

Kidney and prostate	Tissue	Normal	6	[46,47]

Heart and muscle	Tissue	Normal	10	[46,47]

Hematopoietic tissues	Tissue and purified cells	Normal	30	[39,46,47]

Uterus	Tissue	Normal	6	[46,47]

Placenta	Tissue	Normal	3	[46,47]

**Highly cycling cells compendium (n = 22)**				

Leukemia cell lines	Cell line	Malignant	8	[47]

Lymphoma cell lines	Cell line	Malignant	4	[47]

Early erythroid cells	Cultured primary cells	Normal	2	[47]

Endothelial cells	Cultured primary cells	Normal	2	[47]

Hepatocarcinoma cell line	Cell line	Malignant	2	This study

Colorectal cancer cell line	Cell line	Malignant	2	[47]

Breast cancer cell line	Cell line	Malignant	2	This study

A summary of the microarray samples used in this study (A full list of samples is found in Additional file 1)

### A strong cell cycle signature

**Figure 2:**
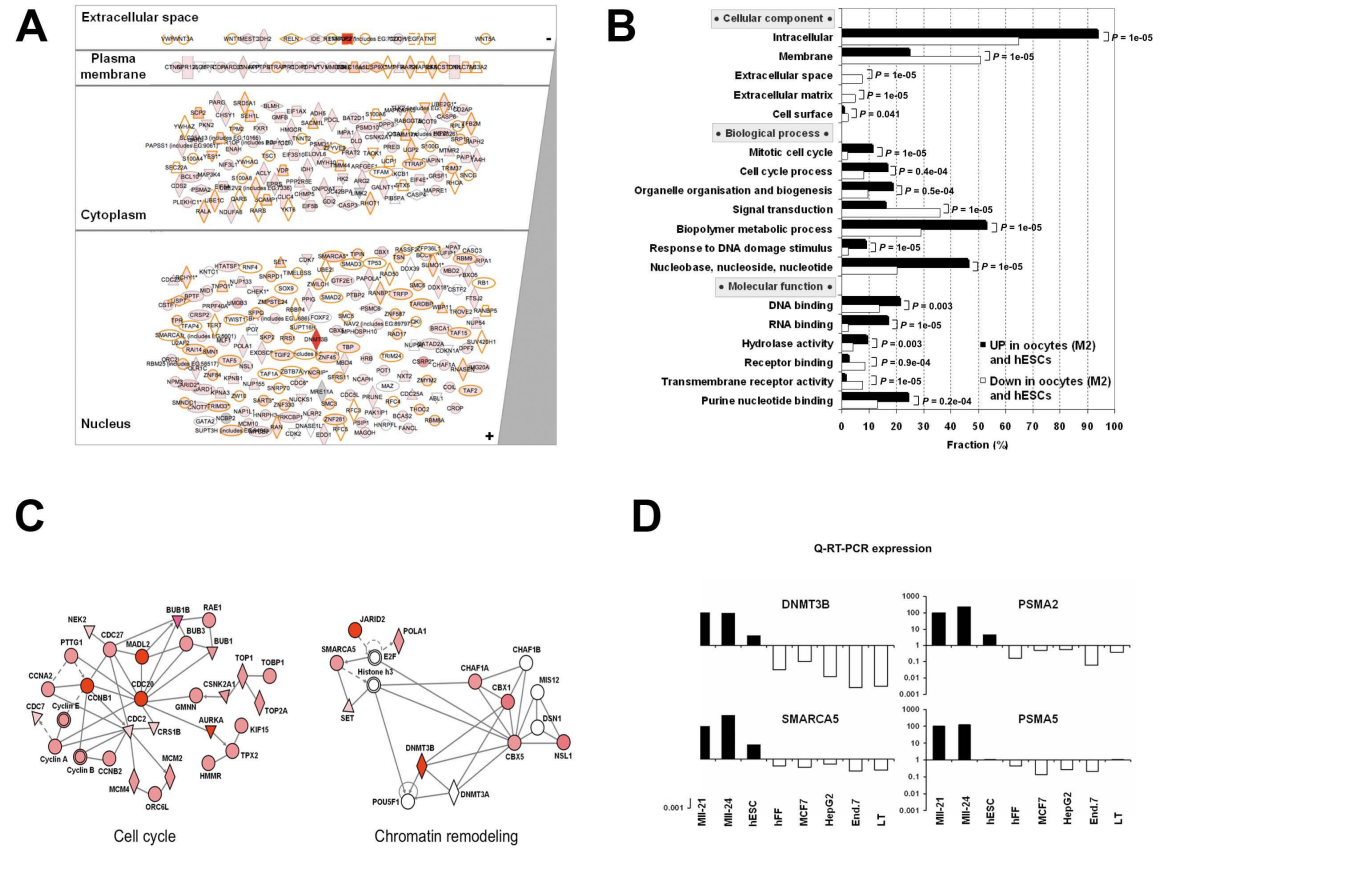
**Biological annotations of genes overexpressed in human mature MII oocytes and hESC**. Cellular compartment localisation according to Gene Ontology (GO) annotations (A). Statistical comparison of the distribution of GO annotations in the oocytes/hESC signature with the genes underexpressed in oocytes and hESC (B). Gene Ontology categories which differed significantly (*p *value ≤ 0.01) between oocytes and hESC are shown. Oocyte/hESC gene networks (C). We computed interaction networks from the oocyte/hESC signature. Genes included in the oocyte/hESC signature are in red (the color intensity is proportional to the oocyte/hESC to somatic samples fold change). Genes not found in the signature are in white. In each network, edge types are indicatives: a plain line indicates direct interaction, a dashed line indicates indirect interaction; a line without arrowhead indicates binding only; a line with an arrowhead indicates "acts on". Node types represent different types of molecules: diamond, enzyme; square, cytokine; triangle, phosphatase; and circle, other. Double line edge represents a group or a complex. Validation of microarray data (D). Gene expression of DNMT3A, SMARCA5, PSMA5, PSMA2 were assayed with quantitative RT-PCR (QRT-PCR). The expression of these four selected genes was compared in mature oocytes, hESC and various somatic samples: human fibroblasts (hFF), human breast adenocarcinoma cell line (MCF7), human hepatocellular liver carcinoma cell line (HepG2), endometrial cells (End7) and T-lymphocyte cells (TL) using QRT-PCR. All measurements were performed in duplicate in two separate runs. The relative levels of gene expression of target mRNA was normalized against *GAPDH *expression. Fold change values are plotted on a log_10 _scale.

To get an insight into the oocyte/hESC signature, we searched for overrepresented gene ontology (GO) functional annotations. As expected from our previous studies on oocytes transcriptome or hESC transcriptome [18,20], the oocytes/hESC signature was highly enriched in genes associated with intra-cellular localization, DNA and RNA binding, and conversely, it was significantly depleted in genes which encode for secreted proteins or proteins implicated in signal transduction (Figure 2A and 2B). Remarkably, we observed that the biological process annotations related to cell cycle such as "mitotic cell cycle", "cell cycle progress", "nucleobase, nucleoside, nucleotide synthesis" were among the most highly enriched in the oocyte/hESC signature (*P *< 1. 10^-3^). These findings were in line with the very short cell cycle duration of primate ESC [21] and with the fact that MII oocytes samples are pure populations of cells undergoing the second meiotic division, which has many features of mitosis. Thus, these two tissue types highly express genes involved in the process of cell division. To delineate more clearly the cell cycle contribution to the oocyte/hESC signature, we defined a cell cycle signature independently from the oocytes and hESC samples. We compared samples characterized by a high proliferation index to our somatic samples series. Proliferating samples included normal cells such as rapidly dividing CD71+ early erythroid progenitors and CD105+ endothelial cells, as well as cell lines originating from haematological, hepatic, breast and colorectal tumors (See Table 1 and Additional file 1 for the list of additional samples). A SAM analysis with a FDR of 0.05% identified 818 PS (682 different Refseq transcripts) overexpressed in proliferating somatic samples, composing a "cell cycle signature". Intersection with the oocyte/hESC signatures revealed that 220 PS (33.7%) were shared with the cell cycle signature (Figure 1D; see Additional file 4). This cell cycle part of the oocytes/hESC signature included enzymes involved in general cell metabolism (*METAP2*, *SHMT2*, etc.), nucleoside synthesis (*DHFR*, *TYMS*, *RRM2*, *PPAT*, etc.), DNA repair including mismatch repair (*MSH2 *and *MSH6*) or base excision repair (*UNG*, *PCNA*), main components of the cell cycle regulatory machinery (*CCNB1 *and *2*, *CCNA*, *CCNE*, etc.), regulator of the topologic state of DNA (*TOP1*, *TOP2A*) and components of the mitotic spindle assembly checkpoint (the centromer constituents *CENPE*, the securin *PTTG1*, and *MAD2L1*, *BUB1B*, *BUB3*) (Figure 2C). However, it must be noted that 36 genes from the oocyte/hESC signature that are functionally annotated "cell cycle" by Gene Ontology were not included in the cell cycle signature. These are cell cycle genes that are preferentially expressed in mature oocytes and hESC as compared to other cycling cell types such as malignant cell lines or proliferating primary hematopoietic cells, and included the spindle checkpoint gene CHEK1, FBXO5/EMI1, the cyclin dependent kinase-activating kinase CDK7 and a component of transcription factor IIH, CDK8.

### Human oocytes and hESC share a large chromatin remodelling network

We focused the second part of our analysis on the non-cell cycle part of the oocyte/hESC signature. Removing the 220 cell cycles PS led to the definition of a "non-cell cycle oocyte/hESC signature" that retained 432 PS (384 transcripts). This signature contained many transcripts involved in DNA and histone modifications. One of these transcripts was *DNMT3B*, involved in DNA methylation. The high fold change of *DNMT3B *in hESC and oocytes compared to somatic samples (43.4 and 9.4, respectively) suggests a central role of this DNA methyltransferase in the control of the epigenome of these cells. In addition, several transcripts, comprising *JARID2*, *SMARCA5*, *CBX5*, *CHAF1A *and *CBX1*, were involved in histone modification processes (Figure 2C). We selected two chromatin remodelling genes, *DNMT3B *and *SMARCA5*, and validated by QRT-PCR their preferential expression in oocytes and hESC compared to somatic samples (Figure 2D).

### Zinc finger genes

Mature MII oocytes and hESC overexpressed numerous zinc finger domain genes. The zinc finger motif is a DNA binding domain dependent on a zinc ion, frequently found in transcription factors. The non-cell cycle oocyte/hESC signature was significantly enriched in zinc finger PS : 20 (18 Refseq transcripts) out of 432 PS (4.6%) in the signature compared to 611 out of 22215 PS (2.7%) in the complete list of PS (*P *= 0.018) (Table 2). The expression bar charts for the zinc finger domain gene ZNF84 is shown in Figure 1C and that of all 18 zinc finger domain genes from the non-cell cycle oocyte/hESC list is available as Additional file 5.

**Table 2:** Genes from the mature oocyte/hESC signature with a Zinc finger domain.

**Gene Symbol**	**Probe Set**	**Gene Title**	**Chromosomal Location**	**Fold change Oocyte MII (a)**	**Fold change hESC (b)**
DPF2	202116_at	D4, zinc and double PHD fingers family 2	chr11q13	6.1	2.1

GATAD2A	218131_s_at	GATA zinc finger domain containing 2A	chr19p13.11	9.8	2.8

LOC730051	221963_x_at	Similar to Zinc finger protein 418	chr19q13.43	5.9	2.5

RCHY1	214281_s_at	ring finger and CHY zinc finger domain containing 1	chr4q21.1	5.7	3.3

ZC3H13	212402_at	zinc finger CCCH-type containing 13	chr13q14.12	3.2	2.4

ZC3H15	201595_s_at	zinc finger CCCH-type containing 15	chr2q32.1	3.5	2.3

ZCCHC8	218478_s_at	zinc finger, CCHC domain containing 8	chr12q24.31	4.5	2.6

ZFAND6	221613_s_at	zinc finger, AN1-type domain 6	chr15q25.1	13.8	3.1

ZMYM2	202778_s_at	zinc finger, MYM-type 2	chr13q11-q12	33.1	3.4

ZNF131	221842_s_at	zinc finger protein 131	chr5p12-p11	8.8	2.2

ZNF281	218401_s_at	zinc finger protein 281	chr1q32.1	6.5	3.1

ZNF3	212684_at	zinc finger protein 3	chr7q22.1	2.8	2.1

ZNF330	209814_at	zinc finger protein 330	chr4q31.1-q31.2	3.5	2.4

ZNF508	203322_at	zinc finger protein 508	chr18q23	4.5	3.9

ZNF588	205739_x_at	zinc finger protein 588	chr7q11.2	6.4	2.1

ZNF84	204453_at	zinc finger protein 84	chr12q24.33	3.1	2.5

ZNF93	215758_x_at	zinc finger protein 93	chr19p12	10.3	2.1

ZZZ3	212893_at	zinc finger, ZZ-type containing 3	chr1p31.1	2.1	2.2

List of genes from the oocyte/hESC signature that display a zinc finger domain, with PS number, and fold change (oocyte versus somatic tissues (a) and hESC versus somatic tissues (b).

### Ubiquitination and proteasome

**Figure 3:**
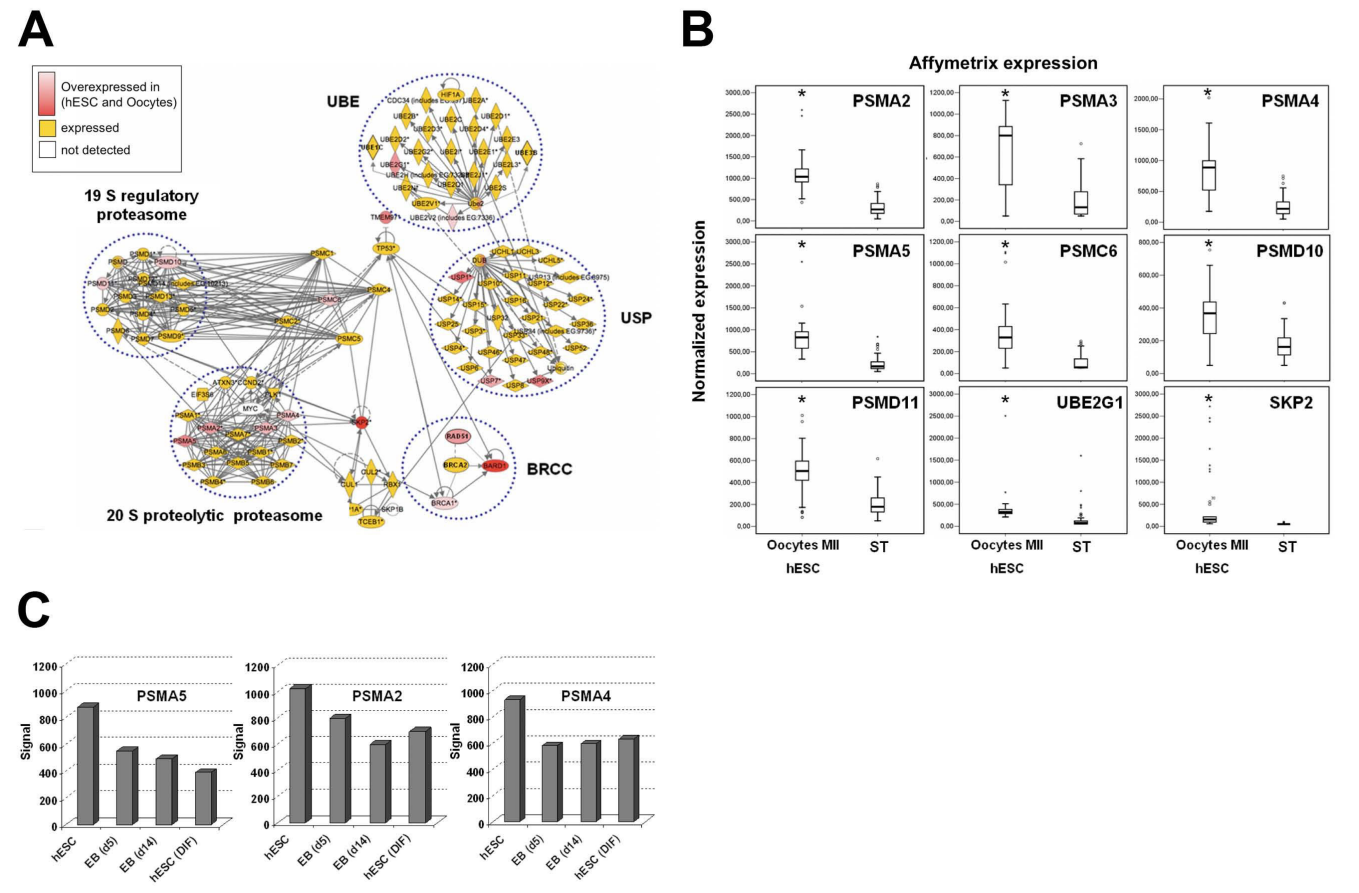
**Overexpression of the ubiquitination/proteasome pathway**. Interaction network analysis of the ubiquitination/proteasome pathway in the oocytes/hESC signature. (A) The interaction network was generated with the Ingenuity software and shows a high number of genes from this cellular pathway overexpressed (red) or expressed (orange) in the oocytes/hESC signature. Transcripts that were not detected by the microarray are white. Notably, we found elements of the ubiquitin-activating enzyme (UBE), BRCA1- and BRCA2-containing complex (BRCC), the regulatory 19S proteasome, the core proteolytic 20S proteasome and the ubiquitin-specific protease (USP) modules in the oocyte/hESC signature. Gene expression measured by microarrays in 9 genes involved in the ubiquitin and proteasome pathway (B). Box-and-whisker plots comparing the expression level of *PSMA2, PSMA3, PSMA4, PSMA5, PSMC6, PSMD10, PSMD11, UBE2G1 *and *SKP2 *in mature oocytes and hESC (38 samples) versus somatic tissues (167 samples). The signal intensity for each gene is shown on the *y *axis as arbitrary units determined by the GCOS 1.2 software (Affymetrix). (*): P-value < 0.0001 using a Mann-Whitney statistical test. Down regulation of the proteasome pathway during hESC differentiation (C). U133A microarray signal values for *PSMA2*, *PSMA4 *and *PSMA5 *in 29 undifferentiated hESC samples (mean value) versus two embryoid bodies (EB) samples (EB day 5 and EB day 14) and 3 non-lineage differentiated hESC samples (mean value).

Surprisingly, we found that a highly significant proportion of genes of the non-cycle oocytes/hESC signature was involved in the protein ubiquitination and proteasome canonical pathway (*P*-value ≤ 1.93E-06, using Ingenuity software) (Figure 3A). Enzymes from the three E1/E2/E3 ubiquitination classes were found significantly overexpressed in the functional regulatory network: the E1 ubiquitin-activating enzyme *UBE1C*, the E2 ubiquitin-conjugating enzymes *UBE2G1*, *UBE2V1 *and *UBE2V2*, and the E3 ubiquitin protein ligases *UBE3B *and breast cancer 1, early onset (*BRCA1*) (Figure 3A). It was recently showed that *BRCA1 *is part of a holoenzyme complex containing *BRCA1*, BRCA2, BARD1 and RAD51 which is called the *BRCA1*- and *BRCA2*-containing complex (*BRCC*) that displays an ubiquitin E3 ligase activity [22]. We found that three out of the four components of *BRCC*, namely *BARD1*, *BRCA1 *and *RAD51 *are overexpressed in MII oocytes and hESC whereas *BRCA2 *is simply expressed. One key consequence of protein ubiquitination is to target proteins for degradation by the 26S proteasome. In line with the biased high expression of ubiquitination pathway components, we also found many subunits of the proteasome significantly overexpressed in MII oocytes and hESC (Figure 3B). As expected for a ubiquitous cell machinery complex, the expression of most subunits of the 26S proteasome is detected by microarrays in oocytes and hESC. Among these, four catalytic alpha proteasome subunits (*PSMA2*, *PSMA3*, *PSMA4 *and *PSMA5*) and three regulatory subunits, the ATPase *PSMC6 *and the non-ATPase *PSMD10 *and *PSMD11*, are significantly upregulated in MII oocytes and hESC as compared to somatic tissues (*P *< 0.001 for each cited proteasome subunit) (Figure 3B), in line with the overexpression of the ubiquitin pathway. In addition, three ubiquitin-specific proteases (*USP1*, *USP7 *and *USP9X*), that are deubiquitinating enzymes, were also found highly expressed in the oocytes and hESC. Remarkably, microarray analysis showed that upon differentiation, the expression level of proteasome components that are overexpressed in hESC decreased to a level similar to that of somatic cells (Figure 3C).

### High sensitivity of human embryonic stem cells to proteasome inhibition

**Figure 4:**
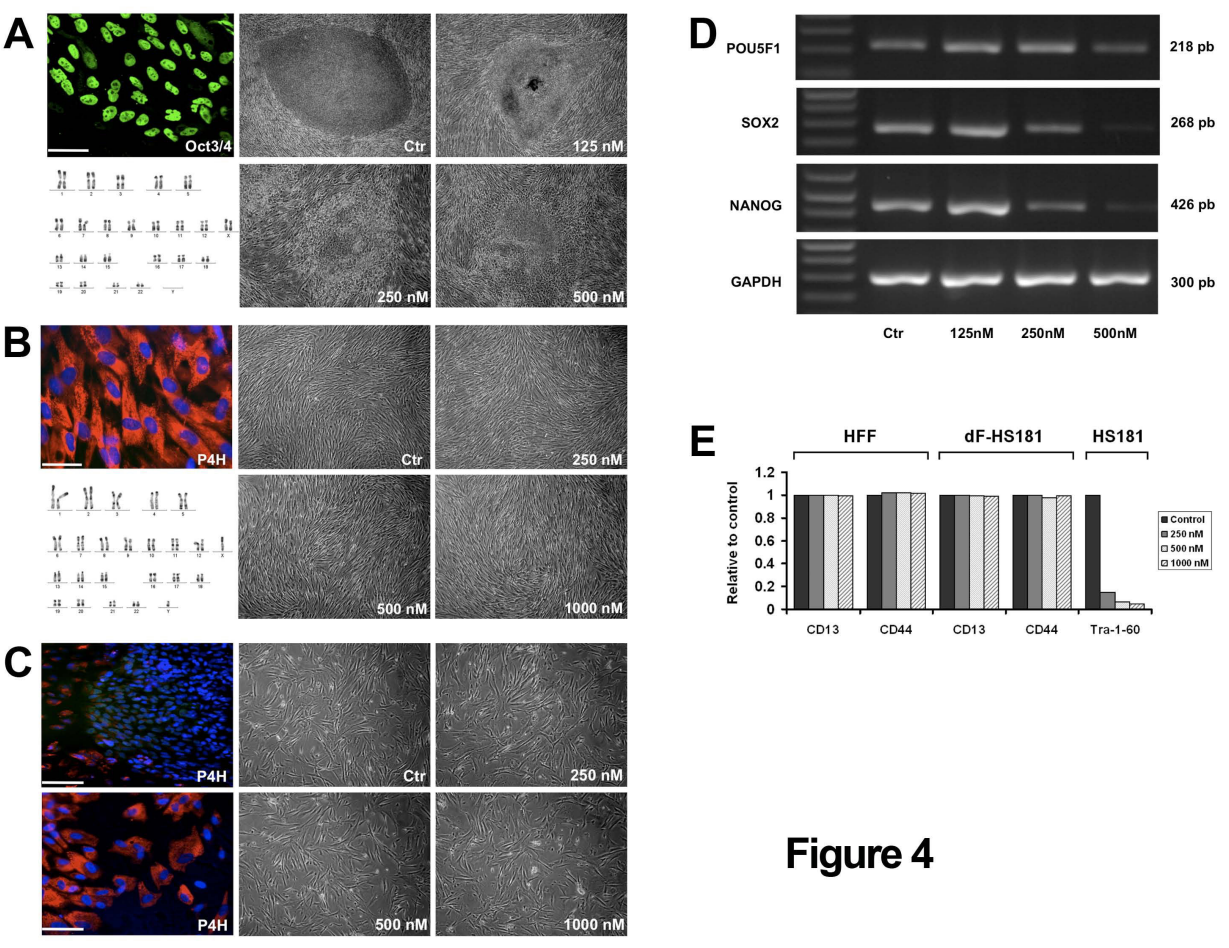
**Blocking proteasome activity in hESC**. Proteasome inhibition causes differentiation of hESCs (A). HS181 colonies (p56) were grown 4 days on hFF and then treated 40 hours with the proteasome inhibitor MG132. Control HS181 colonies express POU5F1 (scale bar 25 μ) and display a normal karyotype. Upon treatment with MG132, HS181 colonies differentiated. No effect of proteasome inhibition on hFF (B). HFF express the fibroblastic marker P4H and display a normal karyotype. HFF were cultured with MG132 during 40 hours. No morphological alteration was observed. No effect of proteasome inhibition on hES-dF-HD90 (C). The HD90 hESC line was differentiated into fibroblasts like cells (hES-dF) that display morphologic feature of dermal fibroblasts and express P4H: bottom left. An undifferentiated colony of HD90 P4H^-^, starts to differentiate at the edges into P4H^+ ^hES-dF (upper left). hES-dF-HD90 cells were cultured with MG132 during 40 hours. No morphological alteration was observed. Down regulation of pluripotency transcription factor in hESC by proteasome blockage (D). The expression of POU5F1/OCT4, NANOG and SOX2 was measured by semiquantitative RT-PCR in HS181 hESC after 40 h culture with MG132. GAPDH was used as a control. Flow cytometry analysis after proteasome inhibition (E). HFF, hES-dF-HS181 and HS181 cells were treated 40 hours with MG132, and then the cell surface fibroblastic markers CD13 and CD44, and the pluripotent cell surface marker TRA-1-60 were measured by FACS. Markers for fibroblastic cells were not altered with MG132 treatment, whereas the marker for pluripotency dropped to barely detectable level. Y-axis: percentage of the control (untreated) sample.

The high expression level of the proteasome machinery in hESC and its decrease during hESC differentiation suggested that this pathway could play an important role in pluripotent cells. To determine the consequence of functional blocking of the proteasome activity in hESC, we tested the effect of MG132, a specific proteasome inhibitor, on embryonic stem cells [23]. Increasing doses of MG132 were added to the culture media of HS181 hES cells. At 250 nM the morphology of the hESC colonies clearly showed large patches of differentiation and at 500 nM no undifferentiated cells remained (Figure 4A). Furthermore, when colonies contained mixed populations of undifferentiated and differentiated cells, 1 μM of MG132 induced the detachment of the undifferentiated cells whereas the differentiated progeny stayed tightly sticked to the dish. These results were reproduced on two other hES cell lines (HD83 and HD90, data not shown). By contrast, the morphology and adhesion of human foreskin fibroblasts (HFF) were not altered by MG132 at concentration up to 1 μM (Figure 4B). Moreover, we differentiated the HD90 and HS181 hESC into hES-differentiated fibroblasts (dF) that display features characteristic of fibroblasts: flattened cells with elongated nucleus and branching pseudopodia, expression of membrane markers such as P4H, CD13 or CD44. Treatment with MG132 did not induce changes in cell morphology of the hES-dF-HD90 (Figure 4C) and hES-dF-HS181 (data not shown), even at high concentrations. RT-PCR analysis showed that expression of pluripotency markers decreased when hESC were treated with MG132. Whereas the expression of POU5F1/OCT4, SOX2 and NANOG decreased with the proteasome treatment, GAPDH expression was not modified (Figure 4D). This effect was also substantiated by flow cytometry. We observed decreasing expression of the pluripotency marker TRA-1-60 on HS181 hESC after exposition to MG132 (Figure 4E). However, high concentration of MG132, up to 1 μM, did not affect two different fibroblasts cell surface markers, CD44 and CD13 on hFF, on hES-dF-HS181 or on hES-dF-HD90. Thus, our results showed that pluripotent hESC are highly susceptible to the action of proteasome blockage, whereas somatic cells such as hFF or the differentiated progeny of hESC such as hES-dF were not affected.

## Discussion

Early human embryo development results in the reprogramming of highly specialized germinal cells into totipotent and then pluripotent cells that are the progenitors of all the specialized cell types of the human body. This unique biological property has been harnessed to restore pluripotency in human somatic cells by SCNT or cell-fusion using embryonic stem cells [6,7]. hESC share with pluripotent stem cells from the inner cell mass pluripotency transcription factors and multi-lineage differentiation properties, and are considered a good *in vitro *model for pre-embryo pluripotent stem cells. Though human oocytes and ESC are developmentally separated by less than one week, the transcriptome of the oocyte undergoes rapid changes after fertilization [24,25]. We undertook to find out a common expression signature to these two cell types, that share somatic cell reprogramming properties, by comparing them to a large collection of somatic tissues samples. A first observation was that the oocytes/hESC signature was highly enriched in genes involved in cell cycle. Whereas this was expected because of the cell cycle status of these two cell types, the expression of a large set of genes associated with cell division is nevertheless of interest for cellular reprogramming. As recently reported, prior mitotic remodeling of the somatic nuclei, involving topoisomerase II (TOP2)-dependent shortening of chromatin loop domains and an increased recruitment of replication initiation factors onto chromatin, is essential for reprogramming of differentiated nuclei [26]. Strikingly, we found that *TOP2A *was highly up-regulated in both oocytes and hESC. This observation suggests that *TOP2A *could be a major factor in the reprogramming properties of oocytes and hESC by participating in chromatin remodeling. Conversely, the identification of a "cell cycle signature", shared with highly proliferating tissues such as cancer cell lines, provided a mean to identify by subtraction a "non-cycle oocytes/hESC" signature of 432 PS. This signature included transcripts coding for proteins involved in chromatin structure modifications such as DNMT3B, JARID2, SMARCA5 or CBX5 that contribute to the DNA methylation and chromatin remodeling (Figure 2C). Consistent with these observations, hESC display a distinct, permissive, chromatin structure compared with other tissues [27]. Expression of DNA methyl-transferases or several ATP-dependent chromatin remodelling factors are elevated in murine oocytes or ES cells [28,29]. Thus our findings show large similarities between murine and human ES cells, and put forward several genes whose strong overexpression could contribute to the specific chromatin state of hESC.

Another lesson from our transcriptomic approach is that the common oocytes/hESC gene expression profile has a very low number of genes that are either secreted or membrane bound (Figure 2A). This is in line with our previously published data that booth oocytes and hESC "specific genes" are significantly depleted in extracellular signalling components, suggesting that this feature is indeed a common characteristic shared by oocytes and hESC and is not simply due to a lack of overlap [18,20]. Hence, genes specifically shared by oocytes and hESC are largely nuclear proteins. One assumption that can be inferred from these findings is that determinant of pluripotency may be mostly intrinsic factors. This observation converges on a recent model, which proposed that pluripotency is a ground state that is intrinsically self-maintained when protected from extrinsic differentiation stimuli [30].

An unexpected observation was that genes involved in protein ubiquitination and proteasome pathway were also overrepresented in the oocytes/hESC signature. This could be linked to the strong proliferation signature of hESC and oocytes as this pathway is by many way implicated in the regulating the cell cycle [31]. However, the overexpression of the ubiquitination/proteasome pathway was still significant when the cell cycle signature was substracted, suggesting that this pathway could have a role in pluripotent cells in addition to its house keeping or cell cycle functions. In line with these results, we showed a selective sensitivity of hESC to the inhibition of the activity of the proteasome, resulting in loss of pluripotency and cell growth at doses without any detectable effects on differentiated but cycling cells such as primary fibroblasts or hESC derived fibroblast like cells. In addition, it must be stressed that the dramatic effects on hESC pluripotency were observed at doses of the proteasome inhibitor MG132 (0.5 μM) significantly lower than those typically found in the literature (several μM) or in mice ES cells (20 μM) [23]. This observation is highly interesting in light of the recent findings of the role of the proteasome in transcription, especially in hESC. The 26S proteasome consists of a 20S core proteolytic part, capped by a 19S regulatory complex. Specificity of degradation of proteins is mediated in part by poly-ubiquitination of the substrate bound for destruction. Based on early work in yeast, the proteasome is known to interact with chromatin and function at multiple steps in transcription, both through proteolytic and non-proteolytic activities [32]. Recently, Szutorisz et al. reported that the 26S proteasome is assembled on intergenic and intragenic regions in ES cells and act as a transcriptional silencer by blocking non-specific transcription initiation [23]. This mechanism involves the proteolytic activity of the 20S core by degrading non-specific preinitiation complexes, thereby preventing permissive transcription and spreading of the modified chromatin. Our results are consistent with this hypothesis, but final answer on this issue will require further investigations.

This work has compared human MII oocytes and hESC to somatic tissues gene expression profiles. One goal was to provide new hints on the process of nucleus reprogramming which takes place in vivo during early embryo development or in vitro during SCNT, and may thus help to improve the iPS technology. Indeed, since the seminal work of the team of Shinya Yamanaka, numerous improvement have been made, including the identification of new genes able to replace some of the original ones in the reprogramming cocktail, the use of small molecules or the replacement of the retroviral vectors by adenoviruses or plasmids [33-37]. A first observation is that human mature oocytes do not express the pluripotency core transcriptional genes *POU5F1/OCT4*, *NANOG *and *SOX2 *[13], except *POU5F1/OCT4 *at low level (see Figure 1C and our Amazonia! on-line expression atlas, ). They neither express *KLF4 *nor *CMYC*, which compose, with *POU5F1/OCT4 *and *SOX2*, the four factors that can reprogram somatic cells by virus-mediated overexpression [11]. From the six "reprogramming" factors described to date, only LIN28 was found in the oocyte/hESC signature. However, POU5F1/OCT4, NANOG, SOX2, KLF4, LIN28 and CMYC are all expressed by hESC. Therefore, during early embryo development, the expression of these genes is induced. Thus, two different molecular pathways that can reprogram adult somatic cells can be envisioned: (i) the process taking place in the oocyte cytoplasm, able to activate the core transcriptional genes, or (ii) the overexpression of the core transcriptional genes themselves together with adjuvant genes, either by viral overexpression or by fusion with cells already expressing these genes. It can be speculated that the factors that lie upstream of the pluripotency core transcriptional circuitry are expressed as mRNA in mature MII oocytes and are still present at blastocyst stage from which hESC are derived. Thus, the oocytes/hESC signature likely includes these factors, and therefore this information could be highly informative for cell reprogramming. The signature contained numerous transcritption factors, including many zinc finger such as *ZNF84*, several still poorly annotated genes such as *KLHL7*, *MRS2*, or the Selenophosphate synthetase 1 (*SEPHS1*), displayed a strong cell cycle signature, chromatin modification genes, and also many actors of the proteasome pathway. All these genes are candidate genes to improve the efficiency of iPS generation, especially in the light of the recent advances that uses non retroviral vectors but at the cost of lower efficacy.

## Conclusion

Human ESC are not only a very promising source of cells for regenerative medicine, but are also a unique tool to understand early embryo development that can not easily be studied on live embryos because of ethical and technical limits. Our comparison of human mature oocytes and hESC to a large collection of somatic samples helps to understand the early embryo development and pluripotency, and is therefore relevant for therapeutics, including improvement of the pregnancy success rate in IVF and regenerative medicine applications such as those involving cell reprogramming.

## Methods

### Transcriptome compendium

We built an expression compendium by combining U133A and U133 Plus 2.0 (Affymetrix, Santa Clara, USA) microarray data from 11 publications and from our laboratory, totalizing 205 samples (Table 1; see Additional file 1) [18,38-47]. Data were analyzed with the GCOS 1.2 software (Affymetrix), using the default analysis settings and global scaling as first normalization method, with a trimmed mean target intensity value (TGT) of each array arbitrarily set to 100. Data was floored at 50, i.e each value below 50 was set to 50. In order to compare U133A and U133 Plus 2.0 data, we further normalized the data with a rank-based normalization method. This method, "MetaNorm", orders the values of the 22 215 PS of the Affymetrix U133A microarray and allocates a new value to each PS according to its rank, using a unique signal value template (Assou et al., manuscript in preparation). Samples are listed in Table 1 (see Additional file 1), along with references, microarray design and, when available, GEO (Gene Expression Omnibus) dataset number. The dataset is available as Additional file 2 (signal and p-value) and each PS can be individually accessed on our website [20].

### Data analysis and visualization

Principle component analysis (PCA) was performed using ArrayAssist^® ^software (Stratagene, La Jolla, CA, USA) to provide a global view of how the various sample groups were related. Hierarchical clustering was carried out with CLUSTER and TREEVIEW software [48]. PCA and clustering were performed on 10,000 PS with the highest coefficient of variation (CV). Gene expression profiles were identified using two-class Significance Analysis of Microarrays (SAM) method [49] which utilizes a Wilcoxon-test statistic and sample-label permutations to evaluate statistical significance. SAM analysis was applied after data filtering retaining only PS with at least 2 samples with a "Present" call. The False Discovery Rate (FDR), an estimate of the fraction of selective genes, was kept below 5% in all statistical analyses. Gene Ontology annotation analysis was carried out using the Fatigo+ tool at the Babelomics website [50]. Only annotations with a false discovery rate-adjusted *P*-value below 0.05 were considered significant. To uncover functional biological networks, we imported gene expression signatures into the Ingenuity Pathways Analysis (IPA) Software (Ingenuity Systems, Redwood City, CA, USA). Comparison of the frequency of zinc finger domain containing transcript between the non-cell cycle oocytes/hESC signature and the entire U133A microarray was carried out using a Pearson's Chi-squared test with Yates' continuity correction.

### Human mature MII oocytes, fibroblasts and malignant cell lines transcriptome

Unfertilized MII oocytes were collected after informed consent 44 hours post insemination or post microinjection by ICSI as previously published [18,44]. Briefly, Mll oocytes were from couples referred to our center for cIVF (tubal infertility) or for ICSI (male infertility). Mature M2 oocytes were pooled: 16 oocytes for Oocyte_M2_16 sample, 21 for Oocyte_M2_21 and 24 for Oocyte_M2_24, from 6, 8 and 8 patients respectively. Human foreskin fibroblasts cell lines were described previously [20]. MCF7 and HEPG2 were from ATCC and cultured in DMEM medium containing 10% fetal calf serum (FCS). Total RNA was isolated using RNeasy mini kits (Qiagen, Courtaboeuf, France) and quantified using a NanoDrop spectrophotometer (Thermo Fischer, Wilmington, Delaware, USA). Total RNA (100 ng) was used to prepare twice amplified labeled cRNA for hybridization to HG-U133 plus 2.0 GeneChip pangenomic oligonucleotide arrays (Affymetrix, Santa Clara, CA, USA) as previously described [18]. The 9 microarray data obtained in our lab are accessible in US National Center for Biotechnology Information, Gene Expression Omnibus (GEO) through the provisional accession numbers GSE11450 (series), and GSM288886, GSM288885, GSM288883, GSM288882, GSM288880, GSM288878, GSM288877, GSM288876, GSM288812 (samples).

### hES cell culture

The HS181 hES cell line was imported from the Karolinska Institute (Stockholm, Sweden). The HD83/D17/FE07-135-L1 and HD90/D18/FE07-142-L1 hES cell lines were derived in our laboratory from a normal embryo and an embryo that carried an abnormal VHL gene according to preimplantation genetic diagnostic, respectively (De Vos et al. manuscript in preparation). Briefly, derivation of HD83 and HD90 was carried out using mechanical dissociation of the inner cell mass [51]. The culture medium used for hESCs derivation and culture consisted of 80% KO-DMEM, 20% knockout SR, 2 mM L-glutamine, 1% nonessential amino acids, 0.5 mM β-mercaptoethanol (all from Gibco Invitrogen, Cergy-Pontoise, France) and 10 ng/mL of bFGF (Abcys, Paris, France). Passaging was performed mechanically by cutting the colony using a #15 scalpel under the microscope. Human foreskin fibroblasts (HFF), mitotically inactivated using irradiation (40 Gy), were used as feeder cells. HFF cells were cultured in 85% DMEM, 15% FBS. All hESC expressed POU5F1/OCT4, NANOG and TRA-1-60, and were able to differentiate into embryoid bodies that expressed differentiation markers. For proteasome inhibition experiments, hESC were incubated 40 h with various concentration of MG132 (Sigma), with medium renewal at 24 h.

### Production of hES-derived fibroblasts

Briefly, hES cells were mechanically isolated and plated on laminin precoated 6-wells culture dishes (Becton Dickinson, San Jose, CA, USA) in hESC culture medium renewed every day. After 5 days, bFGF was removed, and after three additional days, medium was switched to HFF medium. In these conditions, hES cells differentiated into flattened cells with elongated nucleus and branching pseudopodia forming hESC-derived fibroblasts (hES-dF). The hES-dF were then mechanically isolated and transferred to feeder free 6-wells culture dishes. Subsequent passages were carried out using 0.05% trypsin- EDTA (Invitrogen) every 6 days and cultures upscaled into T75 flasks.

### RT-PCR and quantitative PCR (QRT-PCR)

RT-PCR was carried out on total RNA isolated from hESCs grown in the absence or presence of increasing concentration of MG132. PCR conditions and sequence for each primer are shown in Table 3. PCR products were separated on a 1% agarose gel. Expression of the housekeeping gene GAPDH was used to normalise PCR reactions. For QRT-PCR, approximately 1 μg of linear-amplified, biotin-labelled cDNA was mixed with Assays-on-Demand primers and probes and TaqMan Universal Master Mix according to the manufacturer's instructions (Applied Biosystems, Courtaboeuf, France). Real-time QRT-PCR was performed using the ABI Prism 7000 sequence detection system (Applied Biosystems) and normalized to *GAPDH *for each sample using the following formula in which Ct is cycle threshold: 100/2^ΔΔCt^, where ΔΔCt = ΔCt unknown -ΔCt positive control.

**Table 3:** Primer sequences and conditions used for RT-PCR

**Gene**	**Primer sequences (Forward, Reverse)**	**Cycles**	**MgCl2 (mM)**	**Annealing Tm (°C)**	**Product size (bp)**
NANOG	F: CAAAGGCAAACAACCCACTT R: CTGGATGTTCTGG GTCTGGT	30	1.5	62	426

POU5F1/OCT4	F: GACAACAATGAGAACCTTCA R: TTCTGGCGCCGGTTACAGAA	30	1.5	62	218

SOX2	F: ATGGACAGTTACGCGCACAT R: GACTTGACCACCGAACCCAT	30	1.5	62	268

GAPDH	F: AGCCACATCGCTCAGACACC R: GTACTCAGCGGCCAGCATCG	30	1.5	62	238

### Immunofluorescence and cytometry flow

Cells were fixed with PBS containing 4% paraformaldehyde for 20 minutes at room temperature and blocked with PBS containing 5% normal donkey serum for 30 minutes at room temperature. After blocking, cells were incubated with the appropriate primary antibody against POU5F1/OCT4 (sc-9081, Santa Cruz Biotechnology, Santa Cruz, CA; 1:300) or against proline 4-hybroxylase (P4H) (Dako, Trappes, France; 1:50) for 1 hour at room temperature. Cells were washed three times in PBS and incubated for 1 hour at room temperature with Alexa Fluor^® ^488 donkey anti-Rabbit (A-11034; Molecular Probes; 1:1000) and Alexa Fluor^® ^568 goat anti-mouse antibody (A11019, Invitrogen; 1:400) secondary antibodies for POU5F1/OCT4 and P4H respectively. Hoechst staining was added to first wash (Sigma, 5 μg/ml).

For flow cytometry, cells were harvested by treatment with 0.05% trypsin- EDTA (Invitrogen) and were resuspended in culture media. Cell aliquots were incubated on ice with anti-CD13 MAb conjugated to phycoerythrin (PE) (A07762, Beckman-Coulter, 1:50), anti-CD44 MAb conjugated to fluorescein isothiocyanate (FITC) (clone J-173, Immunotech; 1:50) and Tra-1-60 (90232, Chemicon) or conjugated isotypic controls. Flow cytometry was performed on a fluorescence-activated cell sorter (FACS Scan, Becton Dickinson), and data were analyzed with the Cellquest software (Becton Dickinson).

## Authors' contributions

SA: conception and design, collection and assembly of data, data analysis and interpretation, manuscript writing; DC, ST, VP: collection and assembly of data; OH: provision of study material; BK: design; SH: conception and design, provision of study material, manuscript writing; JDV: conception and design, provision of study material, data analysis and interpretation, manuscript writing, final approval of manuscript. All authors read and approved the final manuscript.

## Supplementary Material

Additional file 1 **Table S1**. **Detailed sample list** Click here for file

Additional file 2 **Table S2. Transcriptome compendium**. A compendium of 205 transcriptomes (signal and detection call). Click here for file

Additional file 3 **Table S3. Hierarchical clustering gene lists**. Transcripts lists from gene clusters a, b & c from Figure 1B. Click here for file

Additional file 4 **Table S4. Main transcript signatures**. Transcripts lists from the signatures ana lyzed in the manuscript: non-cell cycle MII oocytes/hESC signature (1), MII oocytes/hESC signature (2), MII oocyte signature (3), hESC signature (4), cell cycle MII oocytes/hESC signature (5) (Exel file). These lists can be found and datamined on our website Amazonia!  using the "gene list" Amazonia! tool. Click here for file

Additional file 5 **Figure S1. Expression bar charts for zinc finger domain containing genes**. Expression bar charts for 18 transcripts overexpressed in oocytes and hESC and containing a zinc finger domain. These charts have been generated on our online gene expression Atlas Amazonia! . Abbreviations as in Figure 1C. Click here for file
