# Supplementary figures and images for: A gene expression signature shared by human mature oocytes and embryonic stem cells
Source: BMC Genomics. 2009 Jan 8;10:10. doi: 10.1186/1471-2164-10-10 (PMC2628676; doi:10.1186/1471-2164-10-10)

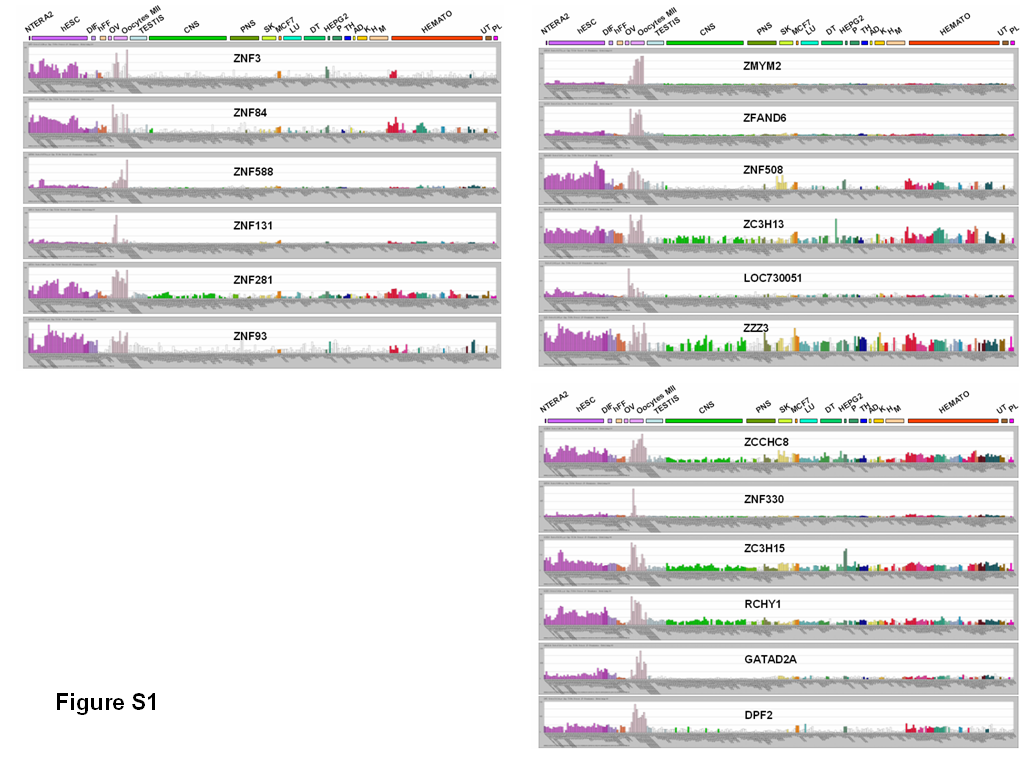

Supplement: Additional file 5 — Figure S1. Expression bar charts for zinc finger domain containing genes. Expression bar charts for 18 transcripts overexpressed in oocytes and hESC and containing a zinc finger domain. These charts have been generated on our online gene expression Atlas Amazonia! . Abbreviations as in Figure 1C. [file 1471-2164-10-10-S5.tiff]
